# NLR, MLR, PLR and RDW to predict outcome and differentiate between viral and bacterial pneumonia in the intensive care unit

**DOI:** 10.1038/s41598-022-20385-3

**Published:** 2022-09-24

**Authors:** Wincy Wing-Sze Ng, Sin-Man Lam, Wing-Wa Yan, Hoi-Ping Shum

**Affiliations:** Department of Intensive Care, Pamela Youde Nethersole Eastern Hospital, 3 Lok Man Road, Chai Wan, Hong Kong SAR China

**Keywords:** Infectious diseases, Respiratory tract diseases, Biomarkers, Diagnostic markers, Predictive markers, Prognostic markers, Microbiology, Bacteria, Pathogens, Virology

## Abstract

The neutrophil-to-lymphocyte ratio (NLR), monocyte-to-lymphocyte ratio (MLR), platelet-to-lymphocyte ratio (PLR), and red cell distribution width (RDW) are emerging biomarkers to predict outcomes in general ward patients. However, their role in the prognostication of critically ill patients with pneumonia is unclear. A total of 216 adult patients were enrolled over 2 years. They were classified into viral and bacterial pneumonia groups, as represented by influenza A virus and *Streptococcus pneumoniae,* respectively. Demographics, outcomes, and laboratory parameters were analysed. The prognostic power of blood parameters was determined by the respective area under the receiver operating characteristic curve (AUROC). Performance was compared using the APACHE IV score. Discriminant ability in differentiating viral and bacterial aetiologies was examined. Viral and bacterial pneumonia were identified in 111 and 105 patients, respectively. In predicting hospital mortality, the APACHE IV score was the best prognostic score compared with all blood parameters studied (AUC 0.769, 95% CI 0.705–0.833). In classification tree analysis, the most significant predictor of hospital mortality was the APACHE IV score (adjusted P = 0.000, χ^2^ = 35.591). Mechanical ventilation was associated with higher hospital mortality in patients with low APACHE IV scores ≤ 70 (adjusted P = 0.014, χ^2^ = 5.999). In patients with high APACHE IV scores > 90, age > 78 (adjusted P = 0.007, χ^2^ = 11.221) and thrombocytopaenia (platelet count ≤ 128, adjusted P = 0.004, χ^2^ = 12.316) were predictive of higher hospital mortality. The APACHE IV score is superior to all blood parameters studied in predicting hospital mortality. The single inflammatory marker with comparable prognostic performance to the APACHE IV score is platelet count at 48 h. However, there is no ideal biomarker for differentiating between viral and bacterial pneumonia.

## Introduction

The complete blood count is frequently used to evaluate sepsis, with focus on the white cell count (WCC) and the presence of left shift or bandaemia^1^. However, an abnormal WCC is not a sensitive marker even in patients with bacteraemia^2^. Although bandaemia is more sensitive for identifying occult bacteraemia^2^, the technical need for manual cell count translates to a substantial delay in diagnosis^3^. The bandaemia response itself is also subject to delay and only emerges one day after clinical infection^1^. These confounding factors have led to a search for more effective markers to aid in the evaluation of infections.

The neutrophil-to-lymphocyte ratio (NLR) is a readily available marker derived from the CBC as a ratio of absolute or relative neutrophil and lymphocyte counts. Endogenous catecholamines and cortisol are released in response to physiological stress, causing an increase in neutrophils and a decrease in lymphocytes^4,5^. Additionally, lymphocyte apoptosis occurs in sepsis, leading to lymphopaenia^6^ and resulting in an elevated NLR. This response promptly occurs within 4 to 8 h of an acute insult^7^, making the NLR superior to leucocytosis or bandaemia for timely reflection of acute illness.

Studies have shown an association between NLR and patient outcomes in septic and bacteraemic patients in the Emergency Department and in the general ward^8–12^, as well as in acute coronary syndrome, acute pancreatitis and rheumatic diseases^13–22^. Its prognostic significance in the intensive care unit (ICU), however, remains uncertain. Similar to the NLR, the monocyte-to-lymphocyte ratio (MLR), platelet-to-lymphocyte ratio (PLR), and red cell distribution width (RDW) are described and evaluated as inflammatory biomarkers in a variety of medical conditions.

The use of NLR may also have diagnostic significance. In bacterial infections, neutrophilia and bandaemia develop, resulting in an increase in NLR. A higher NLR may indicate that an infection is bacterial rather than viral in origin.

Our study aimed to evaluate (1) the prognostic accuracy of these simple biomarkers in predicting hospital mortality compared to the Acute Physiology and Chronic Health Evaluation (APACHE) IV score and (2) their diagnostic power in differentiating pneumonia aetiologies.

## Methods

### Study design and data collection

This retrospective analysis was conducted from January 1, 2017, to June 30, 2019, in Pamela Youde Nethersole Eastern Hospital (PYNEH), a 1700-bed regional hospital in Hong Kong. Patients admitted to the ICU in PYNEH with influenza A or pneumococcal pneumonia were enrolled. Patients with co-infection by both viruses and bacteria, age less than 18 years and insufficient data were excluded. Retrospective analysis of medical records, data in clinical management systems and clinical information systems (IntelliVue Clinical Information Portfolio, Philips Medical, Amsterdam, Netherlands) was performed.

The primary outcome was the ability of NLR to predict hospital mortality. Secondary outcomes were the ability of MLR, PLR and RDW to predict hospital mortality and the diagnostic performance of NLR, MLR, PLR and RDW in discriminating viral from bacterial pneumonia. Laboratory parameters included in the study were obtained from the complete blood count (CBC) at 0 h and 48 h of admission. The delta value was obtained by subtracting the data at 0 h from the data at 48 h.

### Statistical analysis

Demographics, clinical outcomes, and laboratory parameters were compared between hospital survivors and non-survivors and patients with viral and bacterial pneumonia. Categorical variables are expressed as the number of cases and percentages and continuous variables as the median ± interquartile range (IQR). Univariate analysis for categorical variables was performed using Fisher’s exact test or Pearson’s chi-square test, as appropriate. Continuous variables were compared by the Mann‒Whitney U test or Student’s t test. Variables with P < 0.1 in univariate analysis were included in multivariate analysis. Logistic regression analysis with backwards stepwise elimination was used to assess independent predictors for hospital mortality. A P < 0.05 was considered significant.

Comparison of the prognostic and diagnostic accuracy of variables was carried out using receiver operating characteristic (ROC) curves. The area under the receiver operating characteristic curve (AUROC) was calculated, ranging from 0.5 to 1.0. Higher values show greater power in the discriminatory outcome.

A classification tree model was used to identify predictors for hospital mortality. This data mining method classifies the studied population into subgroups of dependent variables based on values of independent variables by using non-parametric testing. The splitting method is called the exhaustive chi-squared automatic interaction detector (CHAID). The analysis was conducted in a stepwise manner using the Pearson chi-squared test. The variable with the smallest Bonferroni-adjusted p value and yielding the most significant split was chosen. Nodes were created that maximised group differences in the outcome. A terminal node was produced when the number of child nodes was below 2 or when the smallest adjusted p value was insignificant. All analyses were performed using Statistical Package for Social Sciences for Windows, version 27.0 (SPSS, Chicago, United States).

The sample size was calculated based on an average AUROC of 0.688 (average AUROC taken from 3 studies: 0.746^23^, 0.695^24^ and 0.622^25^) for the neutrophil-to-lymphocyte ratio (NLR) in predicting hospital mortality. With a type I error of 0.05, power of 80%, and expected mortality rate of 12.5%, the calculated sample size was 189.

### Ethical approval

This study was approved by the Hong Kong East Cluster Ethics Committee of the Hospital Authority (HKECREC-2020-071), which also waived the need for written informed consent due to the retrospective nature of the study. All methods were performed in accordance with relevant guidelines and regulations.

## Results

### Baseline characteristics and clinical outcomes

A total of 216 patients were enrolled during the 2 years indicated. Their baseline characteristics and clinical outcomes are listed in Table 1. The median age was 69 (interquartile range 59–80). The median APACHE IV score was 91 (63–115). The median APACHE IV score for predicting risk of death was 0.39 (0.16–0.66). Most of the population (83.3%, 180/216) had septic shock. Sixty-nine percent (149/216) of patients were mechanically ventilated, and 34.3% (74/216) required renal replacement therapy. The median intensive care unit (ICU) and hospital length of stay were 4 (1.8–9.7) and 13.3 (7.0–28.8), respectively.

**Table 1:** Comparison of patient demographics and outcome parameters between viral and bacterial pneumonia.

	Total (N = 216)	Viral (N = 111)	Bacterial (N = 105)	P value
Age	69 (59 to 80)	69 (55 to 81)	69 (60 to 79)	0.868
Male	129 (59.7)	61 (55.0)	68 (64.8)	0.166
**Co-morbidities**				
Diabetes mellitus	76 (35.2)	48 (43.2)	28 (26.7)	0.015
Hypertension	139 (64.6)	77 (69.4)	62 (59.0)	0.120
Ischaemic heart disease	73 (33.8)	44 (39.6)	29 (27.6)	0.084
COPD	30 (13.9)	16 (14.4)	14 (13.3)	0.846
Liver cirrhosis	3 (1.4)	1 (0.9)	2 (1.9)	0.613
CKD/ESRF	106 (49.1)	58 (52.3)	48 (45.7)	0.345
Solid tumour	6 (2.8)	1 (0.9)	5 (4.8)	0.111
Haematological malignancy	12 (5.6)	4 (3.6)	8 (7.6)	0.242
**APACHE IV**				
Score	91 (63 to 115)	90 (57 to 116)	93 (69 to 114)	0.390
Predicted risk of death	0.39 (0.16 to 0.66)	0.40 (0.13 to 0.67)	0.39 (0.17 to 0.62)	0.833
Presence of septic shock	180 (83.3)	81 (73.0)	99 (94.3)	< 0.001
Mechanical ventilation	149 (69.0)	83 (74.8)	66 (62.9)	0.077
Renal replacement therapy	74 (34.3)	36 (32.4)	38 (36.2)	0.570
**Length of stay (days)**				
ICU	4.0 (1.8 to 9.7)	3.8 (1.7 to 10.7)	4.1 (1.8 to 8.3)	0.807
Hospital	13.3 (7.0 to 28.8)	13.2 (6.8 to 36.1)	13.5 (7.6 to 23.7)	0.851
**Mortality**				
ICU	46 (21.3)	20 (18.0)	26 (24.8)	0.248
Hospital	67 (31.0)	33 (29.7)	34 (32.4)	0.769

*COPD* chronic obstructive pulmonary disease, *CKD/ESRF* chronic kidney disease/end-stage renal failure, *APACHE IV* Acute Physiology and Chronic Health Evaluation IV.

Univariate analysis (Table 1) showed that diabetes mellitus (43.2% vs. 26.7%, P = 0.015) was more common in patients with viral pneumonia. In comparison, patients with bacterial pneumonia were more likely to develop septic shock (94.3% vs. 73%, P < 0.001).

### Comparison between survivors and non-survivors

The overall hospital mortality rate of the enrolled population was 31% (*n* = 67). Univariate analysis (Table 2) showed that hospital non-survivors were older (median 78 vs. 65, P < 0.001) and more likely to have chronic kidney disease or end-stage renal failure (70.1% vs. 39.6%, P < 0.001) and haematological malignancy (10.4% vs. 3.4%, P = 0.051). Hospital non-survivors also had higher APACHE IV scores (110 vs. 79, P < 0.001), APACHE IV score predicted risk of death (0.61 vs. 0.26, P < 0.001), and these patients were more likely to have septic shock (92.5% vs. 79.2%, P = 0.017), received mechanical ventilation (83.6% vs. 62.4%, P = 0.002), required renal replacement therapy (47.8% vs. 28.2%, P = 0.008), and shorter hospital lengths of stay (11.1 vs. 14.1, P = 0.012).

**Table 2:** Comparison between hospital survivors and non-survivors.

	Total (N = 216)	Survivor (N = 149)	Non-survivor (N = 67)	P value
Age	69 (59 to 80)	65 (55 to 77)	78 (68 to 85)	< 0.001
Male	129 (59.7)	85 (57.0)	44 (65.7)	0.294
Viral cause	111 (51.4)	78 (52.3)	33 (49.3)	0.769
**Co-morbidities**				
Diabetes mellitus	76 (35.2)	53 (35.6)	23 (34.3)	0.879
Hypertension	139 (64.6)	100 (67.1)	39 (58.2)	0.222
Ischaemic heart disease	73 (33.8)	47 (31.5)	26 (38.8)	0.351
COPD	30 (13.9)	21 (14.1)	9 (13.4)	1.000
Liver cirrhosis	3 (1.4)	2 (1.3)	1 (1.5)	1.000
CKD/ESRF	106 (49.1)	59 (39.6)	47 (70.1)	< 0.001
Solid tumour	6 (2.8)	5 (3.4)	1 (1.5)	0.668
Haematological malignancy	12 (5.6)	5 (3.4)	7 (10.4)	0.051
**APACHE IV**				
Score	91 (63 to 115)	79 (53 to 103)	110 (93 to 135)	< 0.001
Predicted risk of death	0.39 (0.16 to 0.66)	0.26 (0.11 to 0.52)	0.61 (0.43 to 0.85)	< 0.001
Presence of septic shock	180 (83.3)	118 (79.2)	62 (92.5)	0.017
Mechanical ventilation	149 (69.0)	93 (62.4)	56 (83.6)	0.002
Renal replacement therapy	74 (34.3)	42 (28.2)	32 (47.8)	0.008
**Length of stay (days)**				
ICU	4.0 (1.8 to 9.7)	3.8 (1.7 to 9.5)	4.1 (2.0 to 11.0)	0.508
Hospital	13.3 (7.0 to 28.8)	14.1 (8.0 to 33.4)	11.1 (4.2 to 23.4)	0.012
**WCC**				
0 h	10.7 (6.4 to 15.6)	10.8 (6.8 to 16.3)	10.2 (4.6 to 15.1)	0.232
48 h	11.6 (8.2 to 17.5)	11.4 (8.5 to 17.1)	11.8 (7.3 to 18.1)	0.952
Delta	0.5 (− 2.6 to 5.7)	0.4 (− 2.9 to 5.5)	0.9 (− 1.3 to 6.1)	0.252
**Neutrophils**				
0 h	9.0 (5.4 to 13.0)	9.5 (5.6 to 13.7)	8.4 (4.1 to 11.7)	0.118
48 h	10.3 (7.0 to 16.1)	10.3 (7.2 to 16.0)	10.6 (6.3 to 16.4)	0.719
Delta	0.2 (− 1.5 to 4.8)	0.0 (− 2.1 to 4.4)	0.7 (− 1.0 to 5.5)	0.166
**Lymphocytes**				
0 h	0.7 (0.4 to 1.2)	0.8 (0.5 to 1.2)	0.6 (0.3 to 1.4)	0.058
48 h	0.8 (0.5 to 1.1)	0.9 (0.5 to 1.2)	0.6 (0.3 to 0.9)	0.004
Delta	0.0 (− 0.2 to 0.2)	0.0 (− 0.2 to 0.3)	0.0 (− 0.5 to 0.2)	0.090
**Monocytes**				
0 h	0.4 (0.2 to 0.7)	0.4 (0.2 to 0.6)	0.3 (0.1 to 0.7)	0.155
48 h	0.4 (0.2 to 0.6)	0.5 (0.3 to 0.6)	0.2 (0.1 to 0.6)	0.002
Delta	0.0 (− 0.1 to 0.2)	0.0 (− 0.1 to 0.2)	0.0 (− 0.1 to 0.1)	0.111
**Platelets**				
0 h	171 (121 to 224)	178 (131 to 228)	144 (89 to 206)	0.002
48 h	148 (91 to 197)	164 (117 to 210)	96 (57 to 156)	< 0.001
Delta	− 21 (− 54 to 6)	− 17 (− 47 to 12)	− 31 (− 64 to − 6)	0.018
**NLR**				
0 h	11.6 (5.6 to 18.9)	12.0 (5.7 to 19.9)	10.7 (5.5 to 17.9)	0.694
48 h	14.3 (8.0 to 23.7)	13.4 (7.3 to 22.1)	15.4 (9.4 to 31.4)	0.088
Delta	0.6 (− 3.0 to 8.4)	0.0 (− 3.4 to 5.9)	3.8 (− 1.9 to 11.7)	0.016
**MLR**				
0 h	0.4 (0.2 to 0.8)	0.4 (0.2 to 0.8)	0.5 (0.2 to 0.8)	0.930
48 h	0.5 (0.3 to 0.8)	0.5 (0.3 to 0.8)	0.4 (0.3 to 0.9)	0.403
Delta	0.0 (− 0.2 to 0.2)	0.0 (− 0.1 to 0.2)	0.0 (− 0.2 to 0.3)	0.917
**RDW**				
0 h	14.1 (13.3 to 15.4)	13.9 (13.2 to 15.0)	14.7 (13.7 to 15.8)	0.001
48 h	14.5 (13.7 to 15.6)	14.2 (13.5 to 15.4)	15.1 (14.2 to 16.4)	< 0.001
Delta	0.3 (0.0 to 0.6)	0.2 (0.0 to 0.5)	0.3 (0.0 to 0.6)	0.511
**PLR**				
0 h	231 (132 to 362)	235 (135 to 338)	218 (122 to 419)	0.671
48 h	195 (120 to 294)	200 (126 to 300)	187 (92 to 274)	0.342
Delta	− 23 (− 140 to 53)	− 18 (− 107 to 55)	− 47 (− 203 to 46)	0.163

Delta is defined by the difference between the 0 h and 48 h data (48 h minus 0 h).

*COPD* chronic obstructive pulmonary disease, *CKD/ESRF* chronic kidney disease/end-stage renal failure, *APACHE IV* Acute Physiology and Chronic Health Evaluation IV, *WCC* the white cell count, *NLR* the neutrophil-to-lymphocyte ratio, *MLR* the monocyte-lymphocyte ratio, *RDW* the red cell distribution width, *PLR* the platelet-to-lymphocyte ratio.

Univariate analysis of laboratory parameters (Table 2) showed that lymphocytes at 0 h and 48 h and their delta and that monocytes at 48 h and platelets at 0 h and 48 h and their delta were significantly lower in non-survivors. NLR at 48 h and its delta and RDW at 0 h and 48 h were significantly higher in non-survivors.

### Prognostic performance of laboratory parameters

The prognostic power of the significant parameters identified in univariate analysis was compared with the APACHE IV score, and APACHE IV predicted risk of death by ROC analysis (Table 3). APACHE IV predicted risk of death (AUC 0.776, 95% CI 0.713–0.84), and the APACHE IV score (AUC 0.769, 95% CI 0.705–0.833) had the highest discriminatory ability for the prediction of hospital mortality. Platelets at 48 h performed the best among the laboratory parameters assessed in predicting hospital mortality (AUC 0.721, 95% CI 0.643–0.798).

**Table 3:** Area under receiver operating characteristic curves (AUROC) for prediction of hospital mortality.

	AUROC	SE	95% CI	HL test
APACHE IV risk of death	0.776	0.032	0.713–0.84	0.637
APACHE IV score	0.769	0.033	0.705–0.833	0.055
Platelets 48 h	0.721	0.039	0.643–0.798	0.058
Age	0.711	0.037	0.638–0.784	0.852
RDW 48 h	0.661	0.04	0.582–0.740	0.715
RDW 0 h	0.636	0.041	0.555–0.717	0.716
Platelets 0 h	0.632	0.041	0.55–0.713	0.293
Monocytes 48 h	0.63	0.045	0.542–0.718	0.068
Lymphocytes 48 h	0.621	0.043	0.536–0.706	0.074
Delta NLR	0.603	0.044	0.517–0.688	0.079
Delta platelets	0.601	0.041	0.52–0.682	0.707

*APACHE IV* Acute Physiology and Chronic Health Evaluation IV, *NLR* the neutrophil-to-lymphocyte ratio, *RDW* the red cell distribution width, *AUROC* area under receiver operating characteristic curves, *CI* confidence interval, *HL test* Hosmer‒Lemeshow goodness-of-fit test.

Variables that were associated (P < 0.1) with hospital mortality in the initial univariate analysis (Table 2) were included in multivariate analysis. Table 4 shows the logistic regression analysis of predictors of hospital mortality. Independent predictors of hospital mortality included age (odds ratio 1.052, P = 0.001), APACHE IV score (OR 1.020, P = 0.001), RDW at 48 h (OR 1.268, P = 0.011), delta NLR (OR 1.019, P = 0.051) and platelet count at 0 h (OR 0.994, P = 0.013). The Hosmer‒Lemeshow test was used to ensure the goodness of fit of statistical models, with a p value of 0.814, which indicated good calibration and model fit.

**Table 4:** Logistic regression analysis using backwards stepwise regression (likelihood ratio) for independent predictors of hospital mortality.

	OR	95% CI	P value
Age	1.052	1.022–1.083	0.001
APACHE IV score	1.020	1.008–1.032	0.001
RDW at 48 h	1.268	1.056–1.523	0.011
Delta NLR	1.019	1.000–1.039	0.051
PLT at 0 h	0.994	0.990–0.999	0.013

(a) Factors included within the model building: Age, APACHE IV score, presence of chronic kidney disease, presence of haematological malignancy, presence of septic shock, use of mechanical ventilation, use of renal replacement therapy, lymphocytes at 0 h and 48 h, delta lymphocytes, monocytes at 48 h, platelets at 0 h and 48 h, delta platelets, the neutrophil lymphocyte ratio at 48 h, delta NLR, RDW at 0 h and 48 h (factor selection based on the univariate analysis results from Table 2 with P < 0.1 and without collinearity).

(b) Logistic regression: Hosmer‒Lemeshow test chi-square 4.455, df 8, P = 0.814, AUROC of the model: 0.830, 95% CI 0.772–0.888).

**Figure 1:**
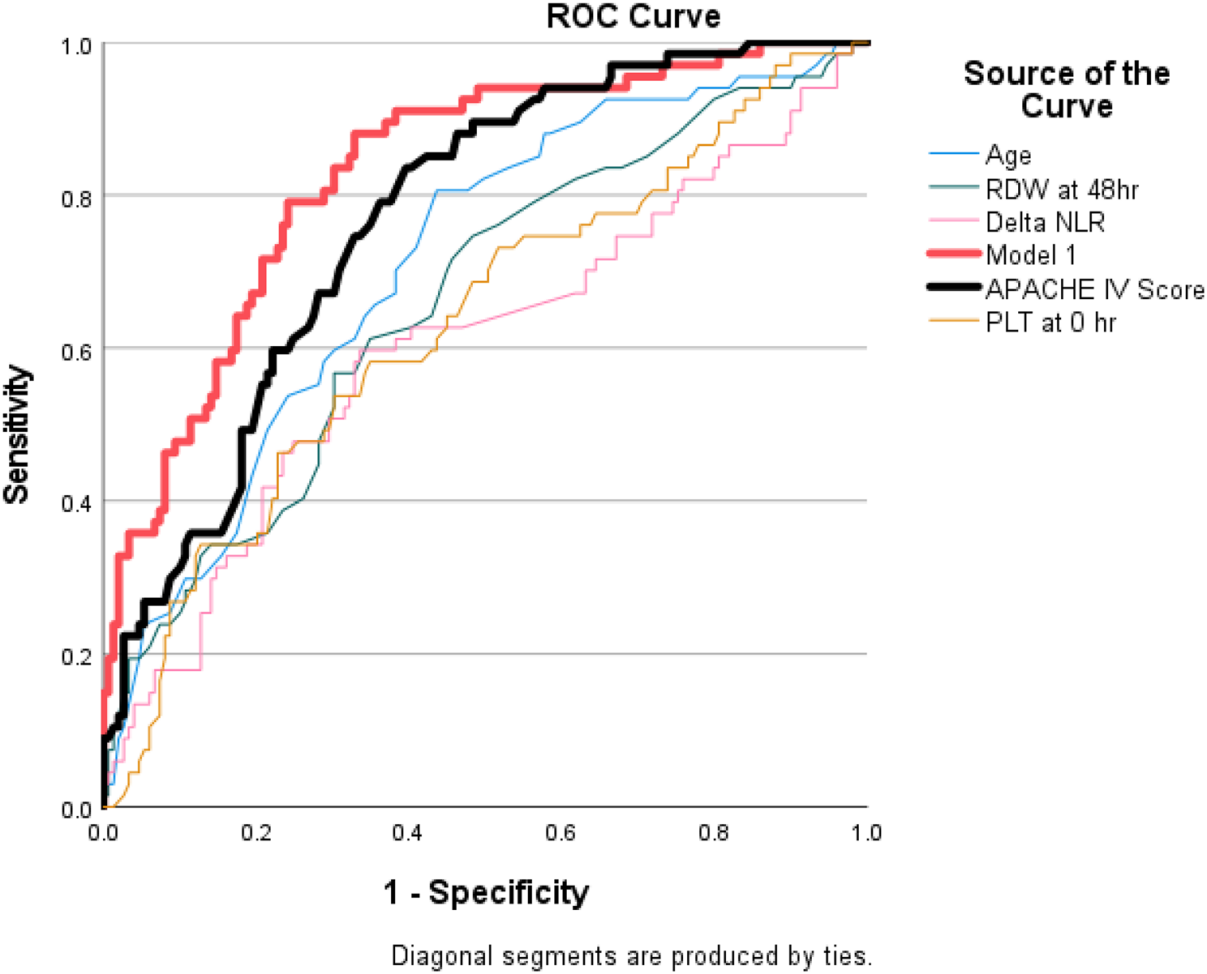
Receiver operating characteristic (ROC) curves to compare the performance of the APACHE IV score and Model 1. Model 1 (logistic regression model from Table 4): AUROC 0.830, 95% CI 0.772–0.888. APACHE IV score: AUROC 0.769, 95% CI 0.705–0.833.

The composite of the 5 parameters in Table 4 was referred to as Model 1. Figure 1 compares the prognostic performance of Model 1 and the APACHE IV score by ROC analysis. Model 1, being a composite of five independent predictors, showed superiority in predicting hospital mortality (AUC 0.830, 95% CI 0.772–0.888) over the APACHE IV score alone (AUC 0.769, 95% CI 0.705–0.833).

### Classification tree analysis

**Figure 2:**
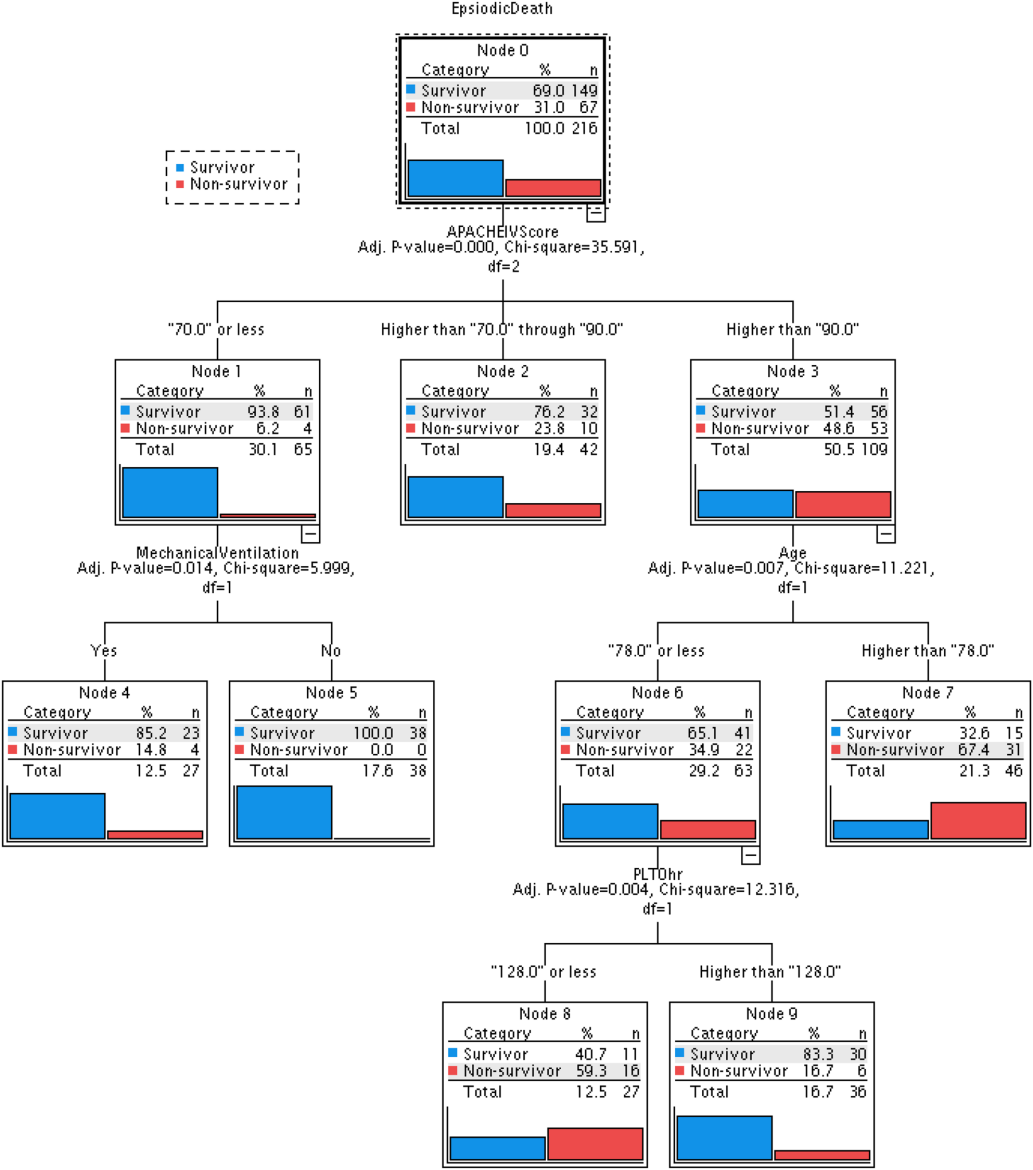
Classification tree analysis for predictors of hospital mortality.

The classification tree model (Fig. 2) was applied to analyse determinant factors that predict hospital mortality. The most significant predictor was the APACHE IV score (adjusted P = 0.000, χ^2^ = 35.591). For patients with APACHE IV scores ≤ 70, those requiring mechanical ventilation had increased hospital mortality (adjusted P = 0.014, χ^2^ = 5.999, hospital mortality rate of 14% vs. 0% compared with those not requiring mechanical ventilation). For patients with APACHE IV scores > 90 and ages > 78 (adjusted P = 0.007, χ^2^ = 11.221), the hospital mortality rate reached 67.4%. For patients with APACHE IV score > 90 and age ≤ 78, platelet count (adjusted P = 0.004, χ^2^ = 12.316) became an important determinant of mortality. Those with platelet counts ≤ 128 at 0 h had higher hospital mortality (59.3% vs. 16.7%) than those patients with platelet counts > 128.

### Diagnostic performance of laboratory parameters

**Figure 3:**
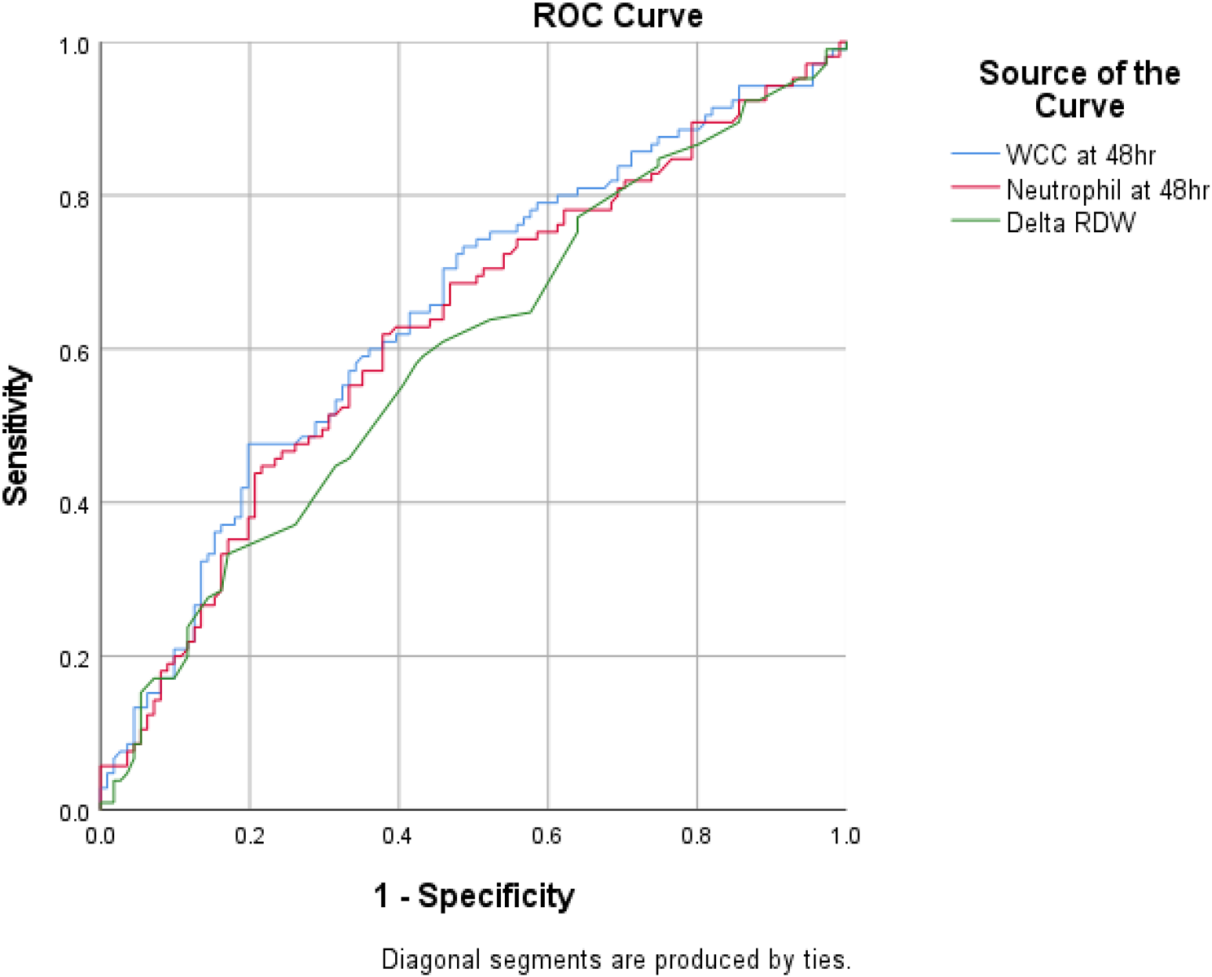
Receiver operating characteristic (ROC) curves to compare the diagnostic performance of laboratory parameters in differentiating viral versus bacterial pneumonia.

Patients with viral pneumonia had a lower white cell count (WCC) at 48 h (9.9 vs. 13.6, P < 0.001), neutrophil count at 48 h (8.8 vs. 11.6, P = 0.001), and delta red cell distribution width (RDW, 0.2 vs. 0.3, P = 0.013) than patients with bacterial pneumonia (Table 5). In receiver operating characteristic (ROC) curve analysis of these parameters (Table 6), WCC at 48 h (AUC 0.648; 95% CI 0.572–0.722) had a greater ability to differentiate viral from bacterial pneumonia than neutrophils at 48 h (AUC 0.627, 95% CI 0.552–0.702) and delta RDW (AUC 0.594, 95% CI 0.518–0.670). Figure 3 displays the respective ROC curves of these parameters.

**Table 5:** Comparison of laboratory parameters between viral and bacterial pneumonia.

	Total (N = 216)	Viral (N = 111)	Bacterial (N = 105)	P value
**WCC**				
0 h	10.7 (6.4 to 15.6)	9.3 (6.2 to 14.7)	11.8 (6.7 to 16.9)	0.093
48 h	11.6 (8.2 to 17.5)	9.9 (7.3 to 14.2)	13.6 (9,7 to 19.4)	< 0.001
Delta	0.5 (− 2.6 to 5.7)	0.6 (− 1.6 to 3.7)	0.4 (− 3.3 to 7.2)	0.351
**Neutrophils**				
0 h	9.0 (5.4 to 13.0)	8.1 (5.4 to 11.7)	9.7 (5.6 to 15.1)	0.084
48 h	10.3 (7.0 to 16.1)	8.8 (6.3 to 12.5)	11.6 (7.9 to 17.4)	0.001
Delta	0.2 (− 1.5 to 4.8)	0.1 (− 0.9 to 3.4)	0.2 (− 2.3 to 6.5)	0.691
**Lymphocytes**				
0 h	0.7 (0.4 to 1.2)	0.7 (0.5 to 1.2)	0.7 (0.4 to 1.2)	0.657
48 h	0.8 (0.5 to 1.1)	0.7 (0.5 to 1.2)	0.8 (0.5 to 1.1)	0.486
Delta	0.0 (− 0.2 to 0.2)	0.0 (− 0.3 to 0.2)	0.0 (− 0.2 to 0.3)	0.184
**Monocytes**				
0 h	0.4 (0.2 to 0.7)	0.4 (0.2 to 0.7)	0.3 (0.1 to 0.6)	0.286
48 h	0.4 (0.2 to 0.6)	0.4 (0.2 to 0.7)	0.4 (0.2 to 0.6)	0.456
Delta	0.0 (− 0.1 to 0.2)	0.0 (− 0.1 to 0.1)	0.0 (− 0.1 to 0.2)	0.734
**Platelets**				
0 h	171 (121 to 224)	164 (116 to 212)	182 (125 to 235)	0.146
48 h	148 (91 to 197)	142 (89 to 188)	156 (92 to 207)	0.477
Delta	− 21 (− 54 to 6)	− 17 (− 47 to 7)	− 26 (− 57 to 4)	0.206
**NLR**				
0 h	11.6 (5.6 to 18.9)	11.0 (4.9 to 17.2)	12.3 (7.0 to 21.9)	0.150
48 h	14.3 (8.0 to 23.7)	13.9 (6.9 to 20.3)	15.1 (8.7 to 28.1)	0.075
Delta	0.6 (− 3.0 to 8.4)	0.0 (− 2.3 to 6.7)	0.9 (− 3.4 to 9.6)	0.517
**MLR**				
0 h	0.4 (0.2 to 0.8)	0.4 (0.2 to 0.8)	0.4 (0.2 to 0.9)	0.755
48 h	0.5 (0.3 to 0.8)	0.5 (0.3 to 0.9)	0.5 (0.3 to 0.8)	0.349
Delta	0.0 (− 0.2 to 0.2)	0.0 (− 0.2 to 02)	0.0 (− 0.2 to 0.2)	0.998
**RDW**				
0 h	14.1 (13.3 to 15.4)	14.1 (13.2 to 15.4)	14.1 (13.4 to 15.4)	0.647
48 h	14.5 (13.7 to 15.6)	14.3 (13.6 to 15.5)	14.7 (13.9 to 15.6)	0.103
Delta	0.3 (0.0 to 0.6)	0.2 (− 0.1 to 0.5)	0.3 (0.1 to 0.7)	0.013
**PLR**				
0 h	231 (132 to 362)	223 (128 to 339)	238 (134 to 446)	0.267
48 h	195 (120 to 294)	196 (119 to 313)	190 (124 to 280)	0.972
Delta	− 23 (− 140 to 53)	− 15 (− 108 to 67)	− 34 (− 188 to 35)	0.107

The delta is defined by the difference between the 0 and 48 h data (48 h minus 0 h).

*WCC* white cell count, *NLR* the neutrophil-to-lymphocyte ratio, *MLR* the monocyte-lymphocyte ratio, *RDW* the red cell distribution width, *PLR* the platelet-to-lymphocyte ratio.

**Table 6:** Area under the receiver operating characteristic curve (AUROC) for diagnostic differentiation of viral versus bacterial pneumonia.

	AUROC	SE	95% CI	HL test
WCC at 48 h	0.648	0.038	0.572–0.722	0.776
Neutrophils at 48 h	0.627	0.038	0.552–0.702	0.782
Delta RDW	0.594	0.039	0.518–0.670	0.211

*HL test* Hosmer‒Lemeshow goodness-of-fit test.

## Discussion

### Neutrophil-to-lymphocyte ratio (NLR) in the prediction of hospital mortality

The NLR has been studied as a marker of severity and prognostication due to its ability to identify states of extreme physiological stress. Its use has been extensive in different diseases and conditions, including rheumatic diseases^13,22^, acute pulmonary embolism^21,26^, acute coronary syndrome^14,20^ and acute pancreatitis^27^. The use of NLR in predicting the severity of community-acquired pneumonia (CAP) has been intensively studied^28^. Its performance was shown to be comparable to the pneumonia severity index (PSI)^29,30^, CURB-65^30^, WCC and CRP^30,31^. Previous studies have proven NLR to be a helpful prognostic marker for patients with sepsis^12,24,32,33^ and, in general, critically ill populations^23,34,35^. However, there are scarce literature on its use in the prognostication of critically ill CAP patients. To the best of our knowledge, our study is the first to explore the use of NLR in pneumonia patients in the ICU setting. We could not demonstrate a significant difference in NLR between survivors and non-survivors in our critically ill cohort. Therefore, NLR may be a useful screening tool to stratify CAP patients before ICU admission but has limited value in prognostication of the critically ill population.

An interesting observation from our study was the use of delta NLR in the prediction of hospital mortality. We detected a significantly higher delta NLR in non-survivors than in survivors (3.8 vs. 0.0, P = 0.016), which resulted from an elevation in NLR from 0 h (median 10.7, IQR 5.5–17.9) to 48 h (median 15.4, IQR 9.4–31.4). This persistent elevation or lack of improvement in NLR indicated treatment failure over the illness trajectory, making it a marker of poor prognosis. Our findings were consistent with previous studies that had similar observations^36,37^.

### Neutrophil-to-lymphocyte ratio (NLR) in the diagnostic differentiation of pneumonia aetiology

NLR has received significant attention for its diagnostic accuracy in sepsis, pneumonia and bacteraemia^7–11,38^. Several studies have proven NLR to be at least a moderate predictor of bacteraemia, with AUROCs ranging from 0.7 to 0.77^8–11^. Compared to other biomarkers, including C-reactive protein (CRP) and procalcitonin (PCT), NLR shows good correlation and comparable performance in diagnosing bacterial sepsis in emergency care settings. In the critically ill population, CRP and PCT appear to be superior to NLR in diagnosing sepsis^39–42^. However, limited literature exists on its use to determine underlying microbiological aetiology. Our study investigated the use of NLR in discriminating between viral and bacterial pneumonia and, to our disappointment, was found to be inferior to WCC. Only 2 paediatric studies have investigated NLR in the differentiation of bacterial and viral pneumonia, consistently demonstrating its poor discriminatory power^43,44^. One possible explanation is that NLR reflects a patient’s physiological stress when critically ill, regardless of microbiological aetiology. To the best of our knowledge, our study is the first to investigate the use of NLR in differentiating between viral and bacterial pneumonia in the adult critically ill population.

### Monocyte-to-lymphocyte ratio (MLR)

Monocytes are leukocytes originating from precursors in the bone marrow that are recruited to inflamed tissues via the bloodstream in response to microbial stimuli. Further differentiation into either macrophages or dendritic cells aids effective microbial clearance at infected sites^45,46^. Mobilisation of monocytes into the peripheral circulation results in an elevated MLR. The MLR has been shown to be useful in the prognostication of rheumatic diseases^47^, malignancies^48^, coronary artery diseases^49^, stroke^50^ and Guillain‒Barre syndrome^51^. Recently, its use in different infections has been investigated, including cellulitis^52^, respiratory virus infection^53^, pneumonia^29,54,55^ and bacteraemia^56^.

The role of MLR as a predictor of clinical outcome has been explored in patients with *Klebsiella* pneumonia^54^, correlating positively with mortality and acting as an independent predictor of severe *Klebsiella* pneumonia, with an AUROC of 0.888 at an optimal MLR cut-off of 0.665. We could not reproduce such a positive correlation between MLR and hospital mortality in our study. The discrepant finding may be explained by the choice of pneumococcus as the representative bacterium in our study, in contrast to *Klebsiella*, a gram-negative organism. The use of MLR was reviewed by Djordjevic et al.^56^, who found significantly higher MLR values in patients with gram-negative blood cultures than in those with gram-positive blood cultures.

MLR can also aid in the diagnosis of bacterial and viral infections. Huang et al*.* reported satisfactory diagnostic performance of MLR in differentiating between patients with community-acquired pneumonia and healthy subjects^29^. Merekoulias et al*.* observed monocytosis, lymphopaenia and hence a reduced lymphocyte-to-monocyte ratio (equivalent to a raised MLR) in outpatients infected by the influenza virus during the H1N1 pandemic^53^. Subsequently, the authors proposed using the lymphocyte-to-monocyte ratio as a screening tool for influenza virus infection, especially at times where the rapid microbiologic test is in great demand.

According to the above studies, MLR may effectively discriminate patients with pneumonia or infected with the influenza virus from healthy subjects. However, its ability to differentiate between the two types of infections is questionable. In our cohort, the monocyte count, lymphocyte count and MLR were not significantly different between the viral and bacterial groups. Hence, MLR did not show significant diagnostic value in distinguishing between viral and bacterial pneumonia. To date, there is no literature on the use of MLR to differentiate different types of pneumonia.

### Platelet (PLT) and platelet-to-lymphocyte ratio (PLR)

Platelets are vital in adaptive immunity and in eliciting an inflammatory response in addition to their primary role in haemostasis^57^. A strong correlation was demonstrated between platelet count and hospital mortality in CAP patients^58–60^. Consistent with previous studies, we showed that non-survivors had significantly lower platelet counts than survivors at both 0 h and 48 h. The predictive performance of platelet count at 48 h (AUROC 0.721) was comparable to the APACHE IV score (AUROC 0.769), with the best performance of all blood parameters in our study.

PLR is increasingly recognised as an indicator of the inflammatory process and has been shown to have good prognostic value in patients with cancers^61^, acute myocardial infarction^15^ or stable coronary artery disease^62^. Its use in prognostication has been extended to the critically ill and septic population, as evidenced by studies showing an association between PLR and ICU length of stay^63^ and even hospital mortality^64,65^. Our study was not able to demonstrate such a correlation between PLR and hospital mortality. The difference in the sample size of the cohorts may be a significant factor contributing to the inconsistent findings.

### Red cell distribution width (RDW)

The red cell distribution width (RDW) measures variability in red blood cell (RBC) size. Significant associations have also been demonstrated between RDW and patients with sepsis and community-acquired pneumonia^66–71^. Several mechanisms have been proposed to explain the correlation between elevated RDW and inflammatory status. Pro-inflammatory cytokines such as interleukin-1β, interleukin-6, and tumour necrosis factor-α have been shown to shorten RBC survival^72^. Erythropoietin production and erythroid precursor cell differentiation are suppressed^73^. Compensatory release of the larger premature RBCs known as reticulocytes into the circulation results in an elevated RDW.

RDW was found to be significantly associated with 30-day mortality when evaluated as a prognostic marker in septic patients at the Emergency Department^69,71^. Our study was able to demonstrate RDW as an independent predictor of mortality in the critically ill population. Apart from the absolute value of RDW, its change from baseline to 72 h after admission was studied in severely septic patients attending the Emergency Department^68^, and it was found to be an independent predictor of hospital mortality. Our study evaluated delta RDW, as defined as the change from baseline to 48 h after admission, and we did not reproduce the result of delta RDW as an independent predictor of mortality. However, to our surprise, delta RDW showed marginal diagnostic ability in differentiating between viral and bacterial pneumonia (AUROC 0.594). To our knowledge, there have been no previous studies on the association between RDW levels and the aetiology of CAP.

### Limitations

Our study has several limitations. First, this was a single-centre study with a limited sample size, affecting the generalisability and reliability of the results. Further studies with larger sample sizes may be helpful. Second, this was a retrospective study and potentially confounded by selection bias. Third, we chose *Streptococcus pneumoniae* as a representative organism for bacterial pneumonia and influenza A for viral pneumonia. Thus, our results may not represent bacterial and viral pneumonia caused by organisms other than pneumococcus and influenza A. Fourth, we did not include novel biomarkers, such as C-reactive protein (CRP) and procalcitonin (PCT), which have been extensively studied and found to be helpful in prognostication for critically ill septic patients. These markers were not readily available in our hospital at the start of our study period and hence were not incorporated to compare the studied biomarkers. Fifth, the treatment provided for pneumonia may have a positive or negative impact on the value of the haematological markers studied, hence affecting evaluation of the diagnostic efficacy of these markers. Last, we did not exclude patients with an immunocompromised state, for instance, patients on long-term corticosteroid use and those infected with human immunodeficiency virus (HIV). These factors can significantly impact the baseline neutrophil count and, hence, the neutrophil-to-lymphocyte ratio, causing confounding in the interpretation of these biomarkers. Additionally, we included patients with active haematological malignancies (5.6%) in our study. The use of NLR has not been validated in this population, and the results should be interpreted with caution.

## Conclusions

In predicting the outcome of critically ill CAP patients, the prognostic power of the APACHE IV score is superior to all the blood parameters studied. The addition of other factors that are independent predictors of mortality to the APACHE IV score further strengthens its prognostic power. However, the APACHE IV score is limited by the need for multiple clinical and laboratory parameters, which is deemed less convenient than parameters directly derived from a simple complete blood count. In our cohort, the single, simple biomarker with comparable prognostic performance to the APACHE IV score by the ROC analysis was found to be the platelet count at 48 h. Further studies should be carried out to investigate the use of other novel inflammatory markers, such as CRP and PCT, in critically ill patients with pneumonia. The use of multiple, composite biomarkers, including CRP and PCT, instead of single biomarkers, should also be considered, and their predictive power compared with that of the APACHE IV score.

In determining the aetiology of pneumonia in critically ill patients, no single biomarker has good diagnostic accuracy.

## Data Availability

The datasets used and/or analysed during the current study are available from the corresponding author on reasonable request.
